# Longitudinal Trajectories of Participant- and Study Partner-Rated Cognitive Decline, in Relation to Alzheimer’s Disease Biomarkers and Mood Symptoms

**DOI:** 10.3389/fnagi.2021.806432

**Published:** 2022-01-31

**Authors:** Catherine E. Munro, Rachel Buckley, Patrizia Vannini, Carla DeMuro, Reisa Sperling, Dorene M. Rentz, Keith Johnson, Jennifer R. Gatchel, Rebecca Amariglio

**Affiliations:** ^1^Center for Brain/Mind Medicine, Brigham and Women’s Hospital, Boston, MA, United States; ^2^Department of Neurology, Massachusetts General Hospital, Harvard Medical School, Boston, MA, United States; ^3^Center for Alzheimer’s Research and Treatment, Department of Neurology, Brigham and Women’s Hospital, Boston, MA, United States; ^4^Melbourne School of Psychological Sciences, Florey Institute, The University of Melbourne, Melbourne, VIC, Australia; ^5^Massachusetts General Hospital, Boston, MA, United States; ^6^Department of Patient-Centered Outcomes Assessment, RTI Health Solutions, Research Triangle Park, NC, United States; ^7^Department of Radiology, Massachusetts General Hospital, Harvard Medical School, Boston, MA, United States; ^8^McLean Hospital, Belmont, MA, United States

**Keywords:** cognitive concerns, Alzheimer’s disease, amyloid, tau, depression, anxiety, mood, longitudinal

## Abstract

Whereas discrepancies between participant- and study partner-reported cognitive concerns on the Alzheimer’s disease (AD) continuum have been observed, more needs to be known regarding the longitudinal trajectories of participant- vs. study partner-reported concerns, particularly their relationship to AD biomarkers and mood symptomology. Additionally, it is unclear whether years of in-clinic data collection are needed to observe relationships with AD biomarkers, or whether more frequent, remote assessments over shorter periods of time would suffice. This study primarily sought to examine the relationships between longitudinal trajectories of participant- and study partner-rated cognitive decline and baseline biomarker levels [i.e., amyloid and tau positron emission tomography (PET)], in addition to how mood symptomatology may alter these trajectories of concerns over a 2-year period. Baseline mood was associated with longitudinal participant-rated concerns, such that participants with elevated depression and anxiety scores at baseline had decreasing concerns about cognitive decline over time (fixed estimate = −0.17, 95% CI [−0.29 to −0.05], *t* = −2.75, df = 457, adj. *p* = 0.012). A significant interaction between baseline amyloid (fixed estimate = 4.07, 95% CI [1.13–7.01], *t* = 2.72, df = 353, adj. *p* = 0.026) and tau (fixed estimate = 3.50, 95% CI [0.95–6.06], *t* = 2.70, df = 331, adj. *p* = 0.030) levels was associated with increasing study partner concerns, but not participant concerns, over time. The interaction between amyloid and study partner concerns remained significant when utilizing only the first year of concern-related data collection. Overall, these results suggest that frequent, remote assessment of study partner-reported concerns may offer additional insight into the AD clinical spectrum, as study partners appear to more accurately update their concerns over time with regard to pathology, with these concerns less influenced by participants’ mood symptomatology.

## Introduction

Discrepancies between participant- and study partner-reported cognitive decline exist on the preclinical and clinical Alzheimer’s disease (AD) continuum (Amariglio et al., 2015; Vannini et al., 2017; Nuño et al., 2019; Ryan et al., 2019). However, the longitudinal course of these concerns about cognitive decline remains unclear, particularly with regard to their relationships with brain-based AD biomarkers (i.e., cerebral amyloid and tau protein burden) in the preclinical or prodromal stages of disease. By linking the longitudinal trajectories of these concerns with cross-sectional *in vivo* brain pathology, we may be able to detect and identify cognitive changes earlier in the course of the disease in clinical practice to provide more time for the intervention and treatment. Additionally, whereas most dementia clinical trials require study partners for reasons of consent, compliance, and collection data that the participant is unable to provide, the rationale for the requirement of study partners in preclinical AD trials and ongoing involvement of study partners throughout the study is less clear (Nuño et al., 2019). If longitudinal discrepancies exist between participant and study partner concerns and are linked to biomarker data, this could represent an additional, sensitive outcome measure that is more cost-effective and less burdensome to both participants and study staff. One recent longitudinal study found that participant-reported cognitive concerns were significantly associated with progression from cognitively unimpaired to a diagnosis of mild cognitive impairment in amyloid-beta-positive (Aβ+) individuals, whereas study partner-rated cognitive decline was more associated with progression from mild cognitive impairment to dementia in Aβ+ participants (Nosheny et al., 2019). Prior work has linked participant-rated cognitive decline to cerebrospinal fluid (CSF) biomarkers, showing subtle relationships between increased participant-reported cognitive concerns and higher CSF tau levels or lower CSF Aβ levels (Wolfsgruber et al., 2015; Miebach et al., 2019; Espenes et al., 2020). In contrast, other work has found that study-partner report of cognitive decline was more consistently and/or more strongly associated with objective cognition and CSF biomarker burden than participant report (Rueda et al., 2015; Valech et al., 2015; Wolfsgruber et al., 2020). More work needs to be done to fully understand the relationship between longitudinal trajectories of participant- and study partner-rated cognitive decline and *in vivo* cerebral tau burden, as data collection for many longitudinal tau positron emission tomography (PET) studies is ongoing.

In many longitudinal observational studies examining cognitive concerns, assessments occur annually during in-clinic visits over the span of many years. However, recent research has suggested that remote (i.e., delivered *via* online or *via* mail) assessments are both acceptable and feasible for many participants and study partners (Geddes et al., 2020). Remote assessment has not only been shown to be feasible in young, cognitively unimpaired individuals, but also in older individuals with and without neurological and/or psychiatric disorders (D’Arcy et al., 2013; George et al., 2016; Wadsworth et al., 2016; Jacobs et al., 2021; Lavigne et al., 2021). For impaired participants, remote assessment might be preferred to reduce participant and study-partner burden as traveling into clinic becomes more physically challenging. The feasibility of remote assessment raises the question as to whether years of annual, in-clinic assessment are needed to provide valuable data predictive of AD biomarker status, or whether more frequent, remote assessments over shorter time periods are sufficient to observe any relationships present.

Finally, there is a well-documented cross-sectional relationship between cognitive concerns and mood symptomatology, in that individuals with greater mood symptomatology often have more cognitive concerns (Lehrner et al., 2014; Yates et al., 2017). Additional work has shown a consistent relationship between participant-reported depressive symptoms and cognitive concerns, though these participant-rated cognitive concerns are not linked to objective cognitive performance (Zlatar et al., 2018). However, some data suggest that mood symptoms alongside cognitive concerns may impact longitudinal outcomes regarding risk of dementia and/or AD biomarker levels; for example, one group demonstrated that higher Aβ+ burden in cognitively unimpaired older adults was associated with increasing mood symptomatology over time, suggesting that emerging neuropsychiatric symptoms may indicate manifestations of preclinical AD (Donovan et al., 2018). A recent longitudinal study also found that individuals with both depression and subjective cognitive decline were at higher risk for dementia than those with either depression or subjective cognitive decline alone (Wang et al., 2021). Additionally, another group showed that in older individuals unlike younger individuals, depressive symptoms were correlated with cognitive concerns and associated with an increased likelihood of self-rated memory decline the following year (Hill et al., 2020). Given known discrepancies between self- and study partner-reported cognitive concerns, obtaining collateral information may represent valuable data to help accurately identify participants with elevated mood symptoms and cognitive concerns which represent preclinical manifestations of AD pathology, compared to those whose cognitive concerns are more related to preexisting mood conditions. Additionally, it is unclear whether participant depressive symptoms modify the longitudinal trajectories of both participant- and study partner-rated cognitive concerns over time.

This study had several aims to address these gaps in the literature. First, we sought to assess the impact of baseline mood symptomatology (i.e., depression and anxiety) on longitudinal trajectories of both participant- and study partner-rated cognitive decline. We hypothesized that participants with elevated mood symptoms would report greater cognitive decline, whereas study partner ratings would be less impacted by mood symptomatology. For our second aim, we sought to compare longitudinal trajectories of both participant- and study partner-rated cognitive decline to cross-sectional biomarker pathology on PET imaging (i.e., amyloid and tau levels). We hypothesized that study partner report will be more associated with biomarkers longitudinally than participant report. Finally, we wanted to determine whether more frequent assessment over shorter time frames (i.e., 1 year of data collection, or the first four remote sessions completed) would be sufficient to observe any longitudinal relationships present in the full, 2-year dataset.

## Materials and Methods

### Participants

All participants were from the Harvard Aging Brain Study (HABS), a longitudinal observational cohort of cognitively unimpaired individuals aged 65 or older at baseline (Dagley et al., 2017). Inclusion criteria at HABS baseline included a score of 0 on the Clinical Dementia Rating Scale, a score of greater than 25 on the Mini-Mental State Examination, scores above age and education-adjusted cutoffs on the 30-Min Delayed Recall of the Logical Memory Story (Wechsler, 1987; ADNI based cutoffs)^1^, and a score of less than 11 on the Geriatric Depression Scale (GDS; Yesavage et al., 1982) at study entry (no score cutoff criteria were set for subsequent annual GDS scores in the study). Exclusion criteria included history of drug or alcohol abuse, head trauma, or current serious medical/psychiatric illness at the time of recruitment. All HABS participants undergo extensive cognitive testing and multimodal neuroimaging, including PET imaging, every 3 years. Each HABS participant is also required to have a study partner who interacts regularly with the participant and can comment on their cognitive abilities and daily activities. Consistent with other observational studies of cognitively normal individuals, imaging biomarker status is not disclosed to participants or study partners.

**FIGURE 1:**
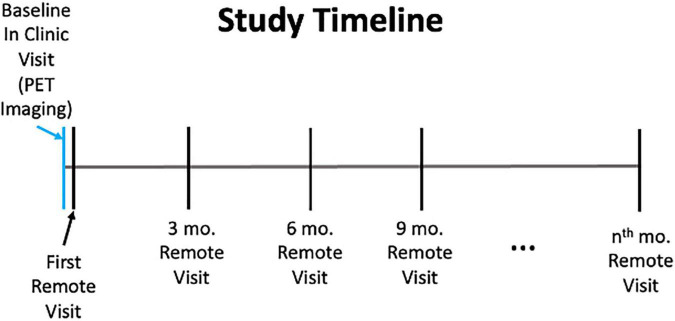
Study timeline.

The analyses presented here utilize data from the Cognitive Function and Mood Study of HABS. The Cognitive Function and Mood Study is a subset of 70 participants (mean age = 76.8, 55.7% women), who are selected from among those HABS participants who were entering a neuroimaging year of the HABS study (Table 1). This subset was demographically generally representative of the overall HABS sample with a slightly smaller percentage of impaired individuals (about 6% impaired in the overall HABS sample with about 4% impaired in this sample), though the Cognitive Function and Mood subset had slightly higher levels of education (overall HABS mean = 15.8 years of education; Cognitive Function and Mood Study mean = 16.7 years of education; *p* = 0.0248). The Cognitive Function and Mood Study was initiated 7 years after the HABS began, and three participants in this sample had progressed to mild cognitive impairment as determined by clinical consensus. Regarding the study timeline, the remote Cognitive Function and Mood Study began with an in-clinic PET imaging visit, after which participants and their study partners were sent questionnaires online *via* REDCap within 1–6 months after their in-clinic visit. Participants and study partners completed additional remote assessments every 3 months thereafter, with a mean of eight sessions completed or about 2 years of assessment in total (Figure 1). Participants had 1 month to complete questionnaires with automatic daily reminders sent out *via* e-mail the first week after they were sent out. Follow-up phone calls were performed by a research assistant as needed if questionnaires were not completed within a week. These REDCap surveys could be completed on any device with access to the Internet and were not restricted to computers. Massachusetts General Hospital Institutional Review Board approval was obtained for both the HABS and the Cognitive Function and Mood substudy prior to study initiation, and informed consent was obtained for both studies from all participants prior to study procedures being performed.

**TABLE 1:** Participant demographics at baseline.

*N* = 70	Mean (SD) [range]
Age	76.8 (6.3) [58–89]
Sex (% F)	55.7
Race (% W)	83
Ethnicity (% NH)	97
Years of education	16.7 (2.6) [12–20]
AMNART VIQ	123.9 (8.2) [90–132]
CDR	0.04 (0.1) [0–0.5]
MCI (n)	3
No. Remote visits completed	7.9 (1.6) [5–10]
Fully completed visits	90%
E4+	27.1%
PIB+ (cutoff of 1.185)	28.1%
FLR PIB DVR	1.2 (0.2) [1.0–1.9]
Entorhinal Tau SUVR	1.1 (0.1) [0.8–1.7]
Geriatric Depression Scale	3.7 (4.3) [0–24]
Geriatric Anxiety Inventory	1.4 (2.7) [0–16]

*F, female; W, White; NH, non-Hispanic; AMNART, American National Adult Reading Test; VIQ, verbal intelligence quotient; CDR, clinical dementia rating; MCI, mild cognitive impairment; E4+, ApoE4-positive; PIB+, Pittsburgh compound B-positive; FLR PIB DVR, frontal, lateral, and retrosplenial Pittsburgh compound B distribution volume ratio; SUVR, standardized uptake ratio.*

### Questionnaires

Questionnaire data were collected and managed using REDCap electronic data capture tools hosted at Massachusetts General Hospital (Harris et al., 2009, 2019). Research electronic data capture (REDCap) is a secure, web-based software platform designed to support data capture for research studies, providing (1) an intuitive interface for validated data capture; (2) audit trails for tracking data manipulation and export procedures; (3) automated export procedures for seamless data downloads to common statistical packages; and (4) procedures for data integration and interoperability with external sources.

The primary measure of interest was a modified version of the cognitive function instrument (aka *Current CFI*; Table 2), comprised of 20 questions regarding current, high-level cognitive functioning using a 5-point Likert scale (i.e., “Never,” “Rarely,” “Sometimes,” “Often,” and “Always”) where a higher score is indicative of greater perceived cognitive decline. This represents an adaptation of the original CFI, which measures participant- and study partner-rated cognitive concerns about change in cognition over the past year over 14 questions and uses a 3-point response scale (i.e., “Yes,” “No,” and “Maybe”; Li et al., 2017). These modifications were made to increase sensitivity and interpretability of data collection and to better capture change in concerns over shorter time periods, as participants were asked about their perceived cognitive decline more frequently than the original CFI. *Current CFI* was administered remotely to both participants and their study partners independently *via* online REDCap surveys every 3 months. A current CFI total score was created for each time point by summing all responses on the 5-point response scale. In terms of compliance, 90% of participants and their study partners fully completed all remote assessments, with the remaining 10% only missing 1–2 remote assessments in total.

**TABLE 2:** Current cognitive function instrument (CFI), participant version.

Please complete these questions thinking about your current ability (most	
recent experience). “Never,” “Rarely,” “Sometimes,” “Often,” “Always”	
1.	How often do you have memory difficulties?
2.	How often do others tell you that you tend to repeat questions over and over?
3.	How often do you misplace things?
4.	How often must you rely on written or electronic reminders (e.g., shopping lists, calendars)?
5.	How often do you forget appointments or family occasions?
6.	How often do you have difficulty remembering important conversations?
7.	How often do you have difficulty recalling names?
8.	How often do you have problems finding the right word when speaking?
9.	How often do you have difficulty with your driving (such as driving more slowly, getting lost, having accidents)?
10.	How often do you have difficulty managing money (such as paying bills, calculating change, doing taxes)?
11.	How often do you turn down invitations for social activities?
12.	How often do you have difficulty with your work performance (paid or volunteer)?
13.	How often do you have difficulty following the news or plots of books, movies, or TV shows?
14.	How often do you have difficulty with your activities (such as hobbies, card games, crafts)?
15.	How often do you become disoriented or lost in familiar places?
16.	How often do you have difficulty using household appliances (such as the washing machine, microwave)?
17.	How often do you have difficulty using electronic devices (such as the cell phone, computer)?
18.	How often do you have difficulty planning an event (such as a dinner party, trip)?
19.	How often do you have difficulty keeping living and work spaces organized?
20.	How often do you have difficulty participating in conversations with a group of friends or family?

*The study partner version of the current CFI is identical to the participant version, with the exception of substituting “your partner” for “your” in the directions.*

Mood was assessed using two scales, the Geriatric Depression Scale (GDS) long form and the Geriatric Anxiety Inventory (GAI; Yesavage et al., 1982; Pachana et al., 2007). The GDS includes 30 yes/no questions designed to measure depressive symptomatology in elderly individuals, with higher scores indicating greater depressive symptoms. On the GDS, scores of 0–9 represent no to mild depressive symptomatology; scores of 11–19 represent mild to moderate depressive symptomatology, and scores of 20–30 represent moderate to severe depressive symptomatology. The GAI is comprised of 20 yes/no questions designed to measure levels of anxiety in elderly individuals, with higher scores indicating greater levels of anxiety. Whereas initial analyses have suggested that a score of 10–11 points indicates significantly elevated levels of anxiety, other studies have found that a score of 8–9 points can adequately detect individuals with an anxiety disorder. The GDS and the GAI were administered in their unmodified forms to participants online *via* REDCap every 3 months.

### Neuroimaging

Magnetic resonance imaging (MRI) was performed on a 3T Tim Trio (Siemens, Washington, DC, United States) and included a magnetization-prepared rapid gradient-echo (MPRAGE) processed with FreeSurfer (FS) as described previously to identify gray-white and pial surfaces to permit ROI parcelation (Braak and Braak, 1997; Delacourte et al., 2002; Fischl et al., 2004; Braak et al., 2006; Becker et al., 2011).

General PET acquisition parameters for HABS have been published previously (Johnson et al., 2016; Dagley et al., 2017). All PET images were acquired on a Siemens ECAT EXACT HR+ scanner. At each time point, PET data were rigidly coregistered to the individual’s closest MPRAGE using SPM12 (Wellcome Department of Cognitive Neurology, Functional Imaging Laboratory, London, United Kingdom). All PET data presented were partial volume corrected using the Müller-Gärtner method, though results were similar when utilizing data that were not partial volume corrected (see Supplementary Material for non-partial volume corrected analyses; Müller-Gärtner et al., 1992).

Cerebral amyloid burden was measured using the Pittsburgh compound B (PIB) radiotracer. PIB-PET images were acquired with a 60-min dynamic acquisition starting directly postinjection. For PIB-PET, distribution volume ratios (DVRs) were calculated *via* Logan plotting with a cerebellar gray reference tissue. Cortical regions of interest were defined from the Desikan–Killiany atlas *via* FreeSurfer v6.0 (Desikan et al., 2006). Frontal, lateral, and retrosplenial (FLR) regions were averaged into a widely accepted global aggregate, as previously reported (Mormino et al., 2014; Johnson et al., 2016; Buckley et al., 2018).

Cerebral tau burden was measured using the Flortaucipir (FTP, formerly known as AV1451) radiotracer, using previously described methods (Johnson et al., 2016). FTP-PET images were acquired approximately 80–100 min after injection. FTP-PET data were examined regionally for these analyses, specifically focusing on bilateral entorhinal cortices (EC) using a FS-defined ROI given the higher likelihood of tau deposition in this region based on a largely cognitively unimpaired sample. FTP binding was expressed in FS ROIs as the SUVR, using the FS cerebellar gray ROI as reference.

### Statistical Analyses

All analyses were conducted in *R* and RStudio, version 4.0.3 (R Core Team, 2019). Linear mixed-effects models were first used to examine potential change in mood (i.e., depression or anxiety measures) and cognitive concerns (both participant- and study partner-reported) over time:

Longitudinal participant or study partner concerns or longitudinal mood ∼ time.

Linear mixed ∼ effects models were also used to assess the interaction between either baseline participant mood (i.e., depression or anxiety measures) or biomarker burden (i.e., amyloid and tau) and time to separately predict longitudinal participant ∼ and study partner ∼ rated cognitive decline:

Longitudinal participant or study partner concerns ∼ baseline amyloid, tau, or mood × time.

In secondary sensitivity analyses, separate linear mixed-effects models were run using a truncated dataset using only the first year of data collection (first four time points for all participants), to examine relationships with biomarkers over a shorter time frame. These models also looked at the interaction between baseline biomarker levels with time to predict longitudinal participant- and study partner-rated cognitive decline, but used only data collected from the first four remote sessions completed. Linear regression models were utilized to observe main effects of the aforementioned models. All models included age, sex, and education as covariates. All *p*-values provided are adjusted using an FDR correction (Benjamini and Hochburg, 1995; Jafari and Ansari-Pour, 2019). Sensitivity analyses were also run, removing subjects with MCI and, for analyses with GDS, removing items related to cognition from the total GDS score.

## Results

### Baseline Mood Symptomatology Predicting Longitudinal Trajectories of Cognitive Concerns

**FIGURE 2:**
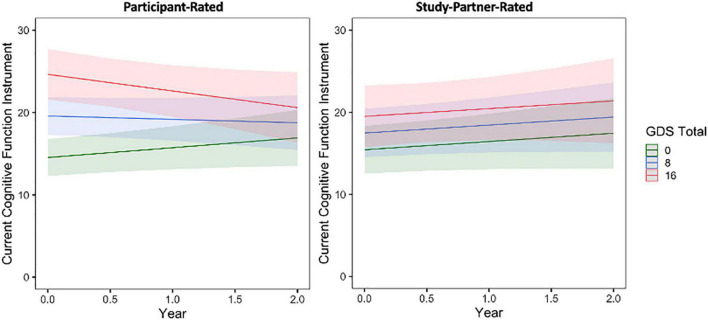
The interaction between baseline participant GDS score and time to predict participant-rated (left) and study partner-rated (right) cognitive concerns using the current CFI. A significant interaction was seen between GDS and time when predicting participant-rated cognitive concerns (fixed estimate = –0.17, adj. *p* = 0.0012), in that participant concerns decreased over time in participants with higher GDS score at baseline (indicative of greater depressive symptomatology). The interaction between GDS score and time predicting study partner-rated cognitive concerns was not significant (fixed estimate = –0.003, adj. *p* = 0.964).

First, regarding longitudinal trajectories of participant-reported mood symptoms over the course of the study, GAI scores were generally stable (slope = 0.01, *t* = 0.04, *p* = 0.9642) whereas GDS scores increased over time, albeit very minimally by about half of a point over each time point (slope = 0.65, *t* = 3.08, *p* = 0.0022). Next, when examining the effects of baseline mood on longitudinal trajectories of cognitive concerns, a significant association was observed between participant-rated cognitive concerns over time and baseline participant GDS score (Figure 2). This interaction was such that individuals with a higher GDS score, indicating greater depressive symptomatology, at baseline had decreasing self-reported cognitive concerns over time (fixed estimate = −0.17, 95% CI [−0.29 to −0.05], *t* = −2.75, df = 457, adj. *p* = 0.012). Results were similar in a sensitivity analyses, using a modified GDS score when items related to cognition or thinking were removed from the GDS total score (fixed estimate = −0.21, 95% CI [−0.36 to −0.07], *t* = −2.84, df = 457, *p* = 0.005) and when individuals with MCI were removed from the sample (fixed estimate = −0.17, 95% CI [−0.29 to −0.04], *t* = −2.54, df = 447, *p* = 0.012). A significant main effect of GDS score was also observed, such that individuals with higher GDS scores tended to have more cognitive concerns at baseline (*t* = 7.83, adj. *p* = 0.004; Figure 2). The interaction between participant GDS score and time was not significant when predicting study partner-rated concerns (fixed estimate = −0.003, 95% CI [1.67–30.38], *t* = −0.05, df = 362, adj. *p* = 0.964), indicating that study partner concerns did not change over time in relation to the level of participant depressive symptomatology reported. These results were similar when using a modified GDS score (removing cognitive items) and when removing individuals with MCI from the sample. Interaction results for predicting study partner-rated concerns were also similar when items related to cognition or thinking were removed from the GDS total score (fixed estimate = 0.04, 95% CI [−0.17 to 0.24], *t* = 0.35, df = 58, *p* = 0.726) and when individuals with MCI were removed (fixed estimate = 0.01, 95% CI [−0.40 to 0.62], *t* = 0.09, df = 352, *p* = 0.927). A significant main effect was seen such that higher participant-rated baseline GDS score, indicating greater depressive symptomatology, was associated with more study partner-rated cognitive decline at baseline (*t* = 2.42, adj. *p* = 0.025). However, this the effect size was smaller compared to that observed in the model predicting participant-rated concerns.

**FIGURE 3:**
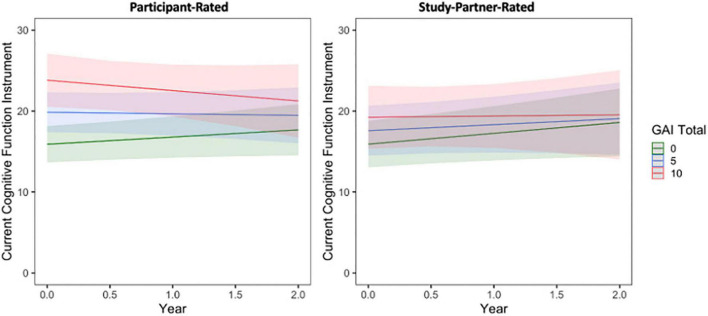
The interaction between baseline participant GAI score and time to predict participant-rated (left) and study partner-rated (right) cognitive concerns using the current CFI. A significant interaction was seen between GAI score and time when predicting participant-rated cognitive concerns (fixed estimate = –0.19, adj. *p* = 0.0450), in that participant concerns decreased over time in participants with higher GAI score at baseline (indicative of greater anxiety symptoms). The interaction between GAI score and time predicting study partner-rated cognitive concerns was not significant (fixed estimate = –0.12, adj. *p* = 0.380).

Similar results were obtained when comparing participant- and study partner-rated concerns to baseline participant GAI score (Figure 3 and see Supplementary Material).

### Baseline Biomarker Levels Predicting Longitudinal Trajectories of Cognitive Concerns

**FIGURE 4:**
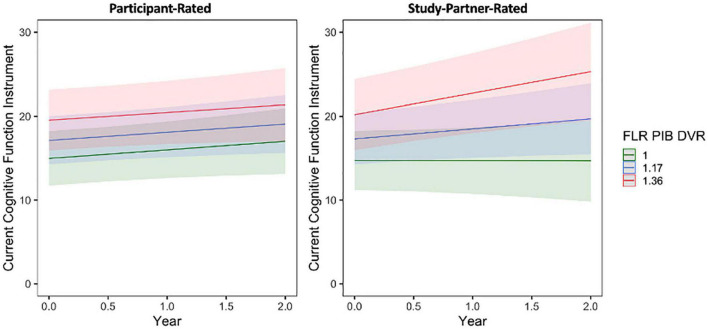
The interaction between baseline participant cerebral amyloid burden (FLR DVR) and time to predict participant-rated (left) and study partner-rated (right) cognitive concerns using the current CFI. A significant interaction was seen between amyloid and time when predicting study partner-rated cognitive concerns (fixed estimate = 4.07, adj. *p* = 0.0260), in that study partner concerns increased over time in participants with higher amyloid burden at baseline. The interaction between amyloid and time predicting participant-rated cognitive concerns was not significant (fixed estimate = 0.44, adj. *p* = 0.6350).

A significant interaction was seen between baseline amyloid level and time when predicting longitudinal study partner-rated cognitive decline (Figure 4), such that a higher participant baseline amyloid burden was associated with increasing study partner concerns over time (fixed estimate = 4.07, 95% CI [1.13–7.01], *t* = 2.72, df = 353, adj. *p* = 0.026). Results were similar when data from participants with MCI were removed from analyses (fixed estimate = 2.95, 95% CI [0.16–5.74], *t* = 2.08, df = 343, adj. *p* = 0.038). A main effect of amyloid was also significant (*t* = 2.40, adj. *p* = 0.026), indicating greater study partner-reported concerns for participants with higher levels of amyloid at baseline. A main effect of amyloid (i.e., higher baseline amyloid levels were related to higher concerns) was also significant in participant ratings (*t* = 2.61, adj. *p* = 0.023; Figure 4), indicating that individuals with higher levels of amyloid had more cognitive concerns at baseline. However, the interaction between amyloid burden and time was not significant when predicting the trajectory of participant-rated cognitive concerns (fixed estimate = 0.44, 95% CI [−1.41 to 2.30], *t* = 0.47, df = 440, adj. *p* = 0.635), suggesting that there was no significant change in participant cognitive concerns over time in relation to baseline amyloid levels.

**FIGURE 5:**
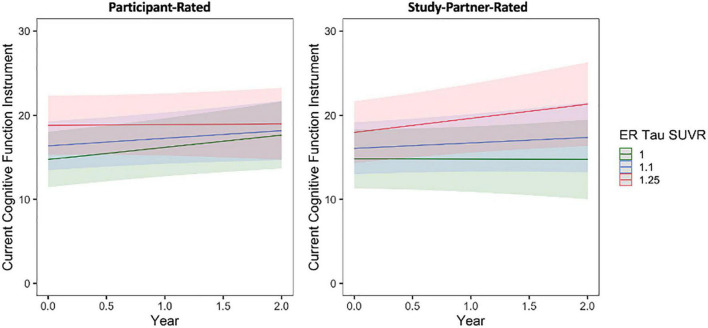
The interaction between baseline participant cerebral entorhinal tau burden (ER SUVR) and time to predict participant-rated (left) and study partner-rated (right) cognitive concerns using the current CFI. A significant interaction was seen between tau and time when predicting study partner-rated cognitive concerns (fixed estimate = 3.50, adj. *p* = 0.030), in that study partner concerns increased over time in participants with higher tau burden at baseline. The interaction between tau and time predicting participant-rated cognitive concerns was not significant (fixed estimate = –1.47, adj. *p* = 0.1620).

A significant interaction was also seen between baseline entorhinal cortex tau burden and time when predicting longitudinal study partner-rated cognitive decline (Figure 5), such that a higher participant baseline tau burden was associated with increasing study partner concerns over time (fixed estimate = 3.50, 95% CI [0.95–6.06], *t* = 2.70, df = 331, adj. *p* = 0.03). This interaction was no longer significant when individuals with MCI were removed from analyses (fixed estimate = 2.09, 95% CI [−0.39 to 4.57], *t* = 1.66, df = 321, adj. *p* = 0.099). A main effect between study partner concerns and baseline participant entorhinal tau burden was marginally significant (*t* = 2.14, adj. *p* = 0.075), trending toward greater study partner-reported concerns for participants who had greater entorhinal tau burden at baseline. When examining participant-rated cognitive concerns, the interaction between entorhinal tau and time (fixed estimate = −1.47, 95% CI [−3.54 to 0.60], *t* = −1.40, df = 409, adj. *p* = 0.162) was not significant (Figure 5). A model examining the association between participant-reported cognitive concerns and entorhinal tau levels at baseline was also non-significant (*t* = 1.73, adj. *p* = 0.120).

Results examining the interaction between baseline tau levels in other temporal lobe regions (i.e., bilateral amygdala and inferior temporal cortex) and time were similar to main analyses and are presented in Supplementary Material, section “Supplementary Biomarker Analyses.”

### Secondary Analyses: Baseline Biomarker Burden Predicting Longitudinal Trajectories of Cognitive Concerns Over Shorter Time Frames

**FIGURE 6:**
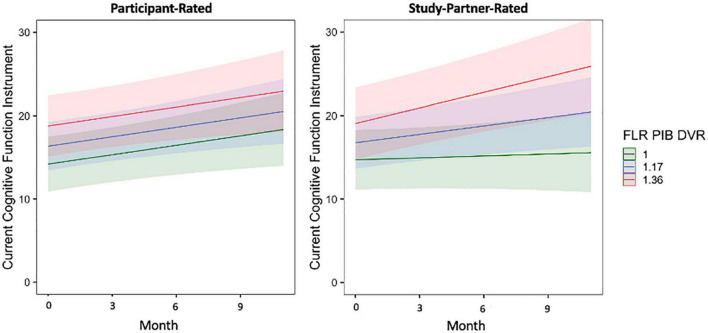
The interaction between baseline participant cerebral amyloid burden (FLR DVR) and time to predict participant-rated (left) and study partner-rated (right) cognitive concerns using the current CFI over only the first year of data collection (first four remote sessions). A significant interaction was seen between amyloid and time when predicting study partner-rated cognitive concerns (fixed estimate = 7.13, adj. *p* = 0.0330), in that study partner concerns increased over time in participants with higher amyloid burden at baseline. The interaction between amyloid and time predicting participant-rated cognitive concerns over the first year of data collection was not significant (fixed estimate = 0.20, adj. *p* = 0.940).

In separate models using participant baseline amyloid and tau burden to predict longitudinal trajectories of participant- and study partner-rated cognitive decline over only the first year of data collection (the first four remote sessions), the interaction between amyloid burden and time in study partner-rated cognitive decline remained significant (fixed estimate = 7.13, 95% CI [1.33–12.92], *t* = 2.43, df = 170, adj. *p* = 0.033; Figure 6). The interaction between entorhinal tau level and time in study partner-rated cognitive decline was not significant when truncating the dataset to only the first four sessions (fixed estimate = 3.38, 95% CI [−1.79 to 8.56], *t* = 1.29, df = 353, adj. *p* = 0.397). Similar to the full dataset, neither the interaction between amyloid and time (fixed estimate = 0.20, 95% CI [−4.92 to 5.31], *t* = −0.08, df = 187, adj. *p* = 0.940) nor the interaction between entorhinal tau and time (fixed estimate = −1.24, 95% CI [−6.81 to 4.32], *t* = −0.44, df = 184, adj. *p* = 0.661) significantly predicted participant-rated concerns.

## Discussion

We observed a significant association between baseline mood symptomatology and participant-rated concerns over time, such that participants with higher depression and anxiety scores at baseline had decreasing cognitive concerns over time. Additionally, there was a strong main effect of mood symptomatology on participant-rated concerns, with higher mood symptoms at baseline associated with more cognitive concerns overall. These results persisted when adjusting models for amyloid and EC tau (see Supplementary Material), suggesting that this finding was not solely driven by greater pathology. Moreover, in a further exploratory analysis, the interaction between baseline mood symptoms and AD biomarkers was not significant in predicting trajectory of participant concerns over time, suggesting that the phenomena observed were not solely driven by participants with greater burden of both mood symptoms and pathology (data not shown). The interaction between participant mood symptoms and time was not seen for study partner-reported concerns, and the main effect of participant mood symptoms on concerns was smaller in study partners. These data indicate that participant-rated cognitive concerns are influenced by their mood symptomatology at baseline, and moreover, that this influence may change over time. Whereas it is still somewhat unclear why participant-rated cognitive concerns decreased over time in those with higher mood symptoms at baseline, this could possibly be explained by enhanced accuracy of participant assessment over time after repeated prompting to reflect on current concerns; results from our sensitivity and exploratory analyses above suggest this is less likely due to participants with greater mood symptomatology at baseline having decreasing awareness of cognitive changes over time. However, future work in larger samples and with longer-term follow-up of the trajectory of mood symptoms and concerns in both participants and study partners is needed to fully differentiate between these alternatives.

Main effects of baseline biomarker burden (i.e., amyloid and tau) predicting both participant- and study partner-reported cognitive concerns were observed, such that higher levels of biomarker burden at baseline were generally associated with greater concerns in both groups. However, we found that higher participant biomarker (i.e., amyloid and tau) levels at baseline were associated only with increasing study partner-rated, but not participant-rated, cognitive concerns over time. These findings are in line with prior research suggesting that, whereas subtle relationships may be seen between participant-reported concerns and biomarkers, there are discrepancies between study partner and participant report that suggest study partner data becomes increasingly valuable as participants progress along the preclinical and clinical AD continuum.

In secondary analyses, the interaction with amyloid remained significant even when utilizing a truncated dataset which included only the first year of data collection (first four remote sessions), suggesting that more frequent remote assessment of study partner concerns may offer additional insight into clinical trajectories over shorter time periods. Additionally, the interaction with amyloid remained significant when individuals with MCI were removed, highlighting that this analysis is sensitive to detect relationships between study partner concerns and amyloid in preclinical individuals. The interaction with tau was not significant using a truncated dataset, indicating a potential power issue (stemming from a small sample size combined with relatively low cerebral tau burden across most participants), or perhaps that more time is needed to observe the relationship between study partner concerns and cerebral tau burden. The fact that the interaction between tau and time predicting study partner-rated cognitive decline lost significance when individuals with MCI were removed seems to provide support for the former explanation, that study partner ratings may be more linked to tau burden in individuals further along the clinical spectrum and may be a good indicator of certain brain pathologies even over shorter time frames.

With regard to the limitations of this study, whereas each participant completed an average of eight remote assessments and compliance with these assessments was strong (90% of participants and study partners fully completed all remote visits), the sample size was relatively small (*n* = 70) and this may have affected our overall ability to observe relationships (i.e., the interaction between tau and time to predict longitudinal study partner-rated cognitive decline) in the truncated dataset of the first four remote sessions. We are also hoping to explore item-level analyses using the current CFI in a larger sample to determine whether there are specific items or factors that may be more predictive of cerebral pathophysiology. Additionally, our sample was largely comprised of cognitively unimpaired adults with relatively low amyloid and/or tau levels and largely subclinical mood symptomatology. Stronger relationships may be observed in samples with more cognitively impaired individuals or individuals with current clinical mood disorders. Additional studies are also needed to explore relationships with tau pathology in other brain regions and consider the impact of mood symptom variability on longitudinal trajectories of cognitive concerns in both participants and study partners. Finally, the lack of racial and ethnic diversity in our highly educated and non-Hispanic or White sample that was slightly more homogenous than the main Harvard Aging Brain Study represents a significant limitation that is seen across many ongoing longitudinal aging studies. Future research studies are needed with participant groups that are more representative of our overall population in terms of racial, ethnic, and socioeconomic diversity to be able to adequately generalize these results.

## Conclusion

Our findings indicated that, over time, study partner rather than participant-reported complaints are more closely associated with participant AD biomarkers and were overall less vulnerable to participant-reported mood symptoms when compared to participants’ ratings of their own cognitive functioning. Moreover, whereas mood symptoms may influence participant-reported concerns, our data suggest that this influence may wane with repeated participant assessment of concerns. This may be in part due to the influence of impaired insight as participants progress along the AD continuum, though more work needs to be done to further investigate this phenomenon using objective cognitive measures and to additionally parse out the specific impact of mood symptomatology over time. Regarding remote data collection, it was demonstrated that frequent, remote assessment of cognitive concerns, particularly with study partners, may offer additional insight into clinical trajectories over shorter periods of time. These findings have implications for both clinical practice and future clinical and observational research studies, highlighting the importance of obtaining longitudinal data from not only participants but also study partners when seeking to identify preclinical or clinical AD.

## Data Availability Statement

The datasets presented in this study can be found in online repositories. The names of the repository/repositories and accession number(s) can be found below: https://habs.mgh.harvard.edu/researchers/.

## Ethics Statement

The studies involving human participants were reviewed and approved by the Institutional Review Board of Mass General Brigham. The patients/participants provided their written informed consent to participate in this study.

## Author Contributions

CM performed the statistical analysis, interpreted the data, prepared figures, and drafted the manuscript. RB performed the statistical analysis, provided assistance or guidance with the interpretation of the data and preparation of figures, and performed critical review of the manuscript. PV and CD performed critical review of the manuscript. RS designed the study and performed critical review of the manuscript. DR performed cognitive assessments and critical review of the manuscript. KJ designed the study and the neuroimaging protocols and performed review of the manuscript. JG performed cognitive assessments, assisted with interpretation of the data, and performed critical review of the manuscript. RA designed the study, performed cognitive assessments, assisted with the interpretation of the data, and performed critical review of the manuscript. All authors contributed to the article and approved the submitted version.

## Conflict of Interest

The authors declare that the research was conducted in the absence of any commercial or financial relationships that could be construed as a potential conflict of interest.

## Publisher’s Note

All claims expressed in this article are solely those of the authors and do not necessarily represent those of their affiliated organizations, or those of the publisher, the editors and the reviewers. Any product that may be evaluated in this article, or claim that may be made by its manufacturer, is not guaranteed or endorsed by the publisher.

## Footnotes

1
http://www.adni-info.org
