# Supplementary material for: Longitudinal Trajectories of Participant- and Study Partner-Rated Cognitive Decline, in Relation to Alzheimer’s Disease Biomarkers and Mood Symptoms
Source: Front Aging Neurosci. 2022 Jan 31;13:806432. doi: 10.3389/fnagi.2021.806432 (PMC8841868; doi:10.3389/fnagi.2021.806432)
Supplement: Supplementary file 1 [file Data_Sheet_1.pdf]

## Supplementary Analyses:

### 1. Supplementary Mood Analyses

A significant interaction was also seen between baseline participant GAI score and time predicting participant-rated cognitive concerns, such that individuals with a higher GAI score at baseline had decreasing cognitive concerns over time (fixed estimate=-0.19, 95%CI[--0.36-(-)0.03], t=-2.29, df=477, adj. p=0.045). Interaction results for predicting participant-rated concerns were marginally significant when individuals with MCI were removed (fixed estimate=-0.15, 95%CI[-0.37-0.17], t=-1.75, df=467, p=0.081). A significant main effect of GAI score was also observed, such that individuals with higher GAI scores tended to have higher cognitive concerns at baseline (t=8.074, adj. p=0.004). The interaction between participant GAI score and time was not significant when predicting study-partner rated concerns (fixed estimate=-0.12, 95%CI-0.38-0.15], t=-0.88, df=375, adj. p=0.380) and there was no significant association between study-partner concerns and baseline GAI score (t=1.650, adj. p=0.139). Interaction results for predicting study-partner rated concerns were similar when individuals with MCI were removed (fixed estimate=-0.10, 95%CI[-0.37-0.17], t=-0.72, df=352, p=0.470).

The interaction observed between participant-rated cognitive concerns over time and baseline participant GDS score remained significant in the model including PIB (fixed estimate=-0.14, 95%CI[-0.26- -0.01], t=-2.12, df=417, p=0.035) as a covariate, but not the model including entorhinal tau (fixed estimate=-0.09, 95%CI[-0.22-0.05], t=-1.26, df=388, p=0.209) as a covariate. The individual predictors of PIB (p=0.3494) and tau (p=0.231) in these models were not significant. Similar to the main analyses reported, the interaction between study-partner-rated cognitive concerns over time and baseline participant GDS score was not significant in models that additionally included PIB (fixed estimate=-0.06, 95%CI[-

0.37-0.17], t=-0.66, df=352, p=0.509) and entorhinal tau (fixed estimate=-0.06, 95%CI[-0.23-0.12], t=-0.64, df=352, p=0.526) as covariates, though both PIB (p=0.01) and entorhinal tau (p=0.012) were significant predictors in these models.

## 2. Supplementary Biomarker Analyses

The analyses for the models using the interaction between non-partial volume corrected biomarker data (i.e., PIB or entorhinal tau) and time to predict longitudinal concerns are summarized in Supplementary Table 1 below.

**Supplementary Table 1:** Analyses using non-partial volume corrected biomarker data. All models are controlling for age, sex, and education.

| Model | Fixed Estimate | 95%CI | t | p |
|---|---|---|---|---|
| Participant concerns ~ PIB*time [Full dataset] | 0.93 | -2.62 – 4.49 | 0.52 | 0.606 |
| Baseline participant concerns ~ PIB [Full dataset; cross-sectional] | --- | --- | 2.10 | 0.040 |
| Study-partner concerns ~ PIB*time [Full dataset] | 7.19 | 1.47 - 12.91 | 2.47 | 0.014 |
| Baseline study-partner concerns ~ PIB [Full dataset, cross-sectional] | --- | --- | 1.77 | 0.081 |
| Participant concerns ~ Entorhinal tau*time [Full dataset] | -4.07 | -8.94 – 0.80 | 1.79 | 0.102 |
| Baseline participant concerns ~ Entorhinal tau [Full dataset, cross-sectional] | --- | --- | 2.21 | 0.031 |
| Study-partner concerns ~ Entorhinal tau*time [Full dataset] | 6.82 | 0.38 – 13.26 | 2.08 | 0.038 |
| Baseline study-partner concerns ~ Entorhinal tau [Full dataset; cross-sectional] | --- | --- | 1.65 | 0.105 |
| Participant concerns ~ PIB*time [1-year dataset] | -0.03 | -9.85 – 9.80 | -0.01 | 0.996 |
| Study-partner concerns ~ PIB*time [1-year dataset] | 15.06 | 4.06 – 26.07 | 2.70 | 0.008 |

| | | | | |
|---|---|---|---|---|
| Participant concerns ~ Entorhinal tau*time [1-year dataset] | -4.61 | -17.93 – 8.70 | -0.68 | 0.495 |
| Study-partner concerns ~ Entorhinal Tau*time [1-year dataset] | 3.71 | -9.14 – 16.56 | 0.57 | 0.570 |

Additional analyses were also run to examine these relationships with bilateral amygdala tau levels and bilateral inferior temporal tau levels. Results were similar to main analyses and are summarized in Supplementary Table 2. Of note, the model examining the interaction between time and baseline amygdala tau levels to predict study-partner rated concerns was marginally significant, unlike analyses involving the entorhinal cortex and inferior temporal cortex which were statistically significant.

**Supplementary Table 2:** Analyses using baseline bilateral amygdala tau and inferior temporal tau levels. All models are controlling for age, sex, and education.

| **Model** | **Fixed Estimate** | **95%CI** | **t** | **p** |
|---|---|---|---|---|
| Participant concerns ~ Amygdala tau*time | -0.79 | -3.23 – 1.65 | -0.63 | 0.526 |
| Study-partner concerns ~ Amygdala tau*time | 1.86 | -0.18 – 6.49 | 1.86 | 0.064 |
| Participant concerns ~ Inferior Temporal tau*time | -2.48 | -5.55 – 0.60 | -1.58 | 0.114 |
| Study-partner concerns ~ Inferior Temporal tau*time | 4.59 | 0.60 – 8.57 | 2.26 | 0.024 |
